# Loss of GCN5 leads to increased neuronal apoptosis by upregulating E2F1- and Egr-1-dependent BH3-only protein Bim

**DOI:** 10.1038/cddis.2016.465

**Published:** 2017-01-26

**Authors:** Yanna Wu, Shanshan Ma, Yong Xia, Yangpeng Lu, Shiyin Xiao, Yali Cao, Sidian Zhuang, Xiangpeng Tan, Qiang Fu, Longchang Xie, Zhiming Li, Zhongmin Yuan

**Affiliations:** 1 Department of Neurosurgery, the Second Affiliated Hospital and Institute of Neurosciences of Guangzhou Medical University, Guangzhou 510260, China; 2 Key Laboratory of Neurogenetics and Channelopathies of Guangdong Province and Ministry of Education of China, Guangzhou 510260, China; 3 Guangdong Province Key laboratory of Brain Function and Disease, Guangzhou 510006, China; 4 Department of Pharmacology, Zhongshan School of Medicine, Sun Yat-sen University, 74 Zhongshan 2nd Road, Guangzhou 510080, China; 5 Department of General Dentistry, 323 Hospital of the People's Liberation Army, Xi'an, China; 6 Department of Radiology, the Second Affiliated Hospital and Institute of Neurosciences of Guangzhou Medical University, Guangzhou 510260, China

## Abstract

Cellular acetylation homeostasis is a kinetic balance precisely controlled by histone acetyl-transferase (HAT) and histone deacetylase (HDAC) activities. The loss of the counterbalancing function of basal HAT activity alters the precious HAT:HDAC balance towards enhanced histone deacetylation, resulting in a loss of acetylation homeostasis, which is closely associated with neuronal apoptosis. However, the critical HAT member whose activity loss contributes to neuronal apoptosis remains to be identified. In this study, we found that inactivation of GCN5 by either pharmacological inhibitors, such as CPTH2 and MB-3, or by inactivation with siRNAs leads to a typical apoptosis in cultured cerebellar granule neurons. Mechanistically, the BH3-only protein Bim is transcriptionally upregulated by activated Egr-1 and E2F1 and mediates apoptosis following GCN5 inhibition. Furthermore, in the activity withdrawal- or glutamate-evoked neuronal apoptosis models, GCN5 loses its activity, in contrast to Bim induction. Adenovirus-mediated overexpression of GCN5 suppresses Bim induction and apoptosis. Interestingly, the loss of GCN5 activity and the induction of Egr-1, E2F1 and Bim are involved in the early brain injury (EBI) following subarachnoid haemorrhage (SAH) in rats. HDAC inhibition not only significantly rescues Bim expression and apoptosis induced by either potassium deprivation or GCN5 inactivation but also ameliorates these events and EBI in SAH rats. Taken together, our results highlight a new mechanism by which the loss of GCN5 activity promotes neuronal apoptosis through the transcriptional upregulation of Bim, which is probably a critical event in triggering neuronal death when cellular acetylation homeostasis is impaired.

Subarachnoid hemorrhage (SAH) is a disastrous cerebrovascular disease, with a combined mortality and disability rate >50%.^1^ Accumulating evidences suggested that neuronal apoptosis is a critical pathological contributor to the SAH-induced early brain injury (EBI), which is considered to be the primary cause of a poor outcome for the patients and defined as the events occurring within the first 72 h after the SAH onset,^2, 3, 4^ but related molecular mechanisms are still not well understood.

Acetylation homeostasis refers to a steady acetylation status of histones and non-histone proteins, resulting from a kinetic balance between histone acetyl-transferase (HAT) and histone deacetylase (HDAC) activities.^5^ The homeostasis confers stability to the cellular homeostasis by coordinating gene expression and repression on both a temporal and spatial basis and therefore has a vital role in modulating cellular fate.^6^

Recently, it was found that the loss of acetylation homeostasis is closely associated with neuronal apoptosis and nervous diseases.^7, 8^ Once the balance becomes impaired, the HDAC:HAT ratio tilts in favour of HDAC activity in terms of availability and enzymatic functionality. Indeed, various HDAC inhibitors have been demonstrated to prevent neuronal apoptosis induced by oxidative stress^9^ or DNA damage.^10^ Furthermore, nervous diseases, including Alzheimer's disease (AD), Parkinson's disease, Huntington's disease, ischaemia, haemorrhage, multiple sclerosis and Friedreich's ataxia, were ameliorated with obvious decreases in apoptosis after administration of HDAC inhibitors.^8, 11^ However, decreasing HAT activity also results in a loss of acetylation; the critical HAT member whose loss of activity contributes to neuronal apoptosis remains to be identified.

General control non-derepressible 5 (GCN5) was the first enzyme identified to possess intrinsic HAT activity and link histone acetylation to transcriptional regulation.^12^ GCN5 exists as components of two large multisubunit complexes, which have global chromatin-modifying functions by acetylating histones genome wide or perform gene-specific regulation through specific loci.^13^ Meanwhile, these complexes also regulate multiple transcriptions through the deubiquitylation of H2B^14^ or suppression of p53-dependent apoptosis by deubiquitinating and stabilizing Sirt1.^15^ GCN5−/− mice die during embryogenesis and is featured with excessive apoptosis.^16, 17^ GCN5 depletion accelerates cerebellar and retinal degeneration in spinal cerebellar ataxia type 7 (SCA7) mice.^18^ These observations suggest that GCN5 has important roles in sustaining the homeostasis of acetylation, deubiquitylation and cellular survival.

In this study, we made an effort to understand the mechanism underlying the regulation of neuronal fate by GCN5. We found that inactivation of GCN5 results in E2F1- and Egr-1-dependent Bim induction and neuronal apoptosis. Furthermore, the loss of GCN5 activity and the transcriptional upregulation of E2F1, Egr-1 and Bim are involved in the SAH-induced EBI in rats. Interestingly, inhibition of HDACs significantly rescues Bim induction, apoptosis and EBI in SAH rats. Our results highlight that GCN5 loss-caused Bim induction is a critical event in triggering neuronal death when the cellular acetylation homeostasis is impaired.

## Results

### Inhibition of GCN5, but not p300 and Tip60, induces neuronal apoptosis

**Figure 1:**
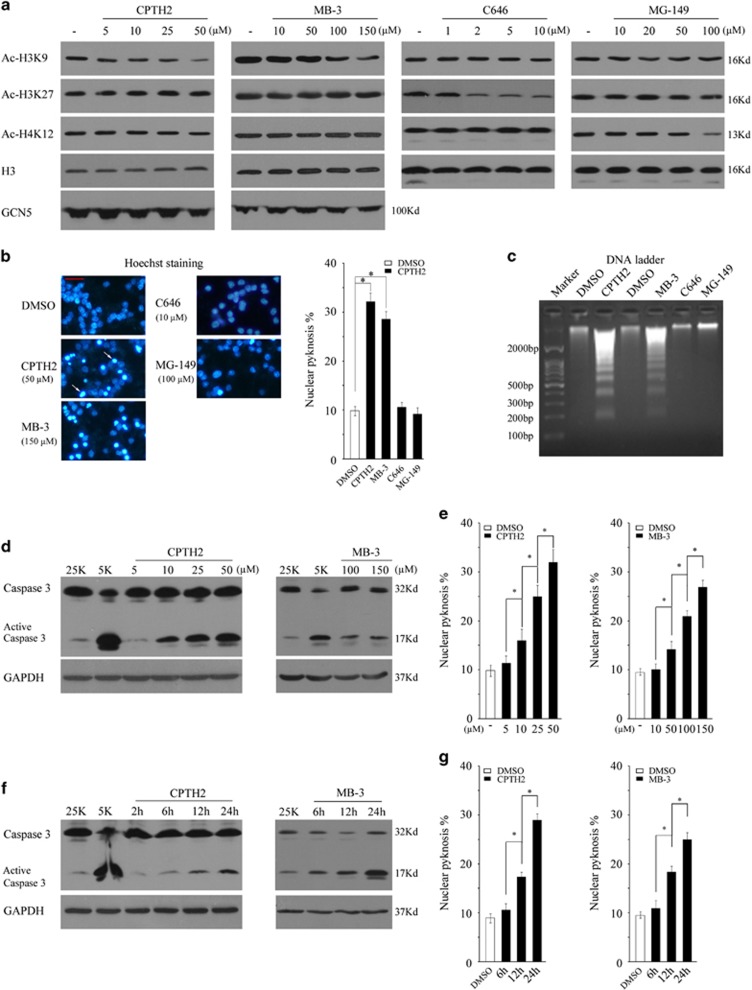
Inhibition of GCN5 activity results in a typical apoptosis. (**a**) DIV 7 CGNs treated with GCN5 inhibitors CPTH2, MB3, p300 inhibitor C646 or Tip60 inhibitor MG149 at the indicated doses for 12 h were subjected to WB to detect the level of Ac-H3K9, Ac-H3K27, Ac-H4K12, H3, H4 or GCN5. (**b** and **c**) CGNs treated with CPTH2 (50 *μ*M), MB-3 (150 *μ*M), C646 (10 *μ*M) or MG-149 (100 *μ*M) in 25K media for 24 h were subjected to nucleic staining or DNA ladder analysis. Fluorescent photos were taken on the Olympus IX71 microscope (scale bar=20 *μ*m). The white arrows indicate the apoptotic cells with nuclear pyknosis. The apoptotic rate was determined by scoring the percentage of cells with nuclear pyknosis in total Hoechst-stained ones. (**d** and **e**) CGNs treated with CPTH2, MB3 in 25K media at the indicated doses for 24 h and the active Caspase 3 by WB and apoptotic rate were determined as in panel (**b**). 5K treatment causes an active Caspase 3 as a positive control. (**f** and **g**) CGNs were treated with CPTH2 (50 *μ*M) or MB-3 (150 *μ*M) in 25K media for the indicated time courses, the active Caspase 3 by WB and apoptotic rate were determined as in panel (**b**). Data were presented as means±S.E.M., *n*=3, *P<0.05. DMSO, dimethyl sulphoxide; GAPDH, glyceraldehyde 3-phosphate dehydrogenase

To determine a HAT member that is critical for neuronal survival, cerebellar granule neurons (CGNs) were treated with GCN5 inhibitors CPTH2 and MB-3,^19, 20, 21^ p300 inhibitor C646 or Tip60 inhibitor MG-149 at different doses for 24 h.^22, 23, 24^ The efficiency of the inhibitors was first evaluated by testing the changes in acetylation of H3K9, H3K27 or H4K12, which were evidenced to be catalysed by GCN5, p300 or Tip60, respectively.^25, 26, 27^ A dose-dependent inhibition of H3K9 acetylation by CPTH2 or MB-3 was observed, without affecting the acetylation of H3K27 or H4K12, or the expression of GCN5, although C646 or MG-149 dose-dependently suppresses the acetylation of H3K27 or H4K12, respectively, without decreasing H3K9 acetylation (Figure 1a). These results suggest that the corresponding inhibitor used shows high efficiency and specificity in blocking the activity of the three HATs.

Next, we assessed whether apoptosis occurs following treatment with the inhibitors by testing the typical apoptotic features, including nuclear pyknosis, DNA laddering and Caspase 3 activation.^28^ CGNs were induced to undergo apoptosis by potassium deprivation (5K treatment) as positive controls. Both CPTH2 and MB-3, but not C646 or MG-149, remarkably induced nuclear pyknosis and DNA laddering (Figures 1b and c, *P*<0.05). Furthermore, CPTH2 and MB-3 dose- and time course-dependently activated Caspase 3, 6 or 7 and increased nuclear pyknosis, but C646 or MG-149 had no such effects (Figures 1d–g, *P*<0.05, Supplementary Figures S1a and b).The results suggest that inhibition of GCN5, but not p300 or Tip60, leads to a typical apoptosis in CGNs.

**Figure 2:**
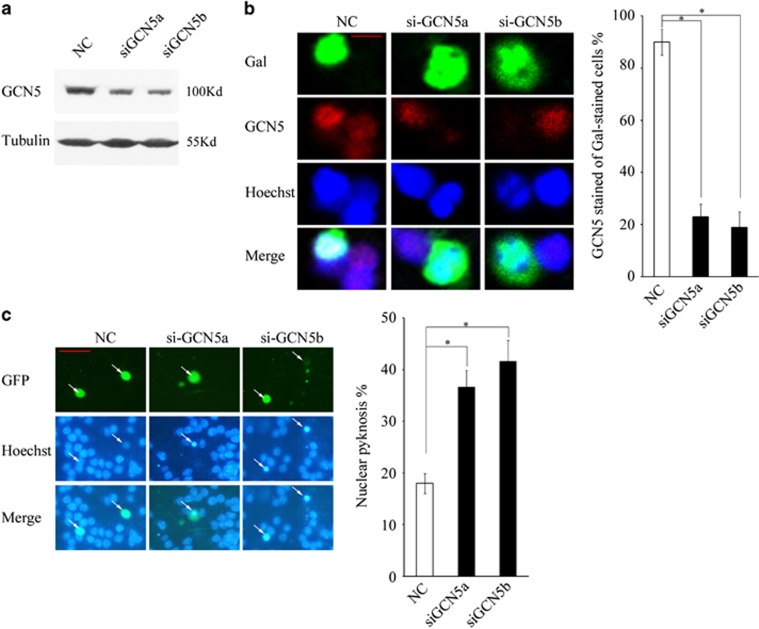
Knockdown of GCN5 by siRNAs induces neuronal apoptosis. (**a**) Rat C6 glioma cells transfected with NC (non-targeted control siRNA), siGCN5a or siGCN5b were subjected to WB to detect the GCN5 expression. Tubulin was reprobed to verify equal loadings. (**b**) DIV5 CGNs transfected with siGCN5a and siGCN5b together with pCMV-Gal plasmids were subjected to immunofluorescence and photos were taken on confocal microscope (scale bar=5 *μ*m). *β*-Gal was stained to mark the transfected cells and the efficiency of RNA interference was determined by scoring the percentage of stained *β*-Gal-positive neuron population with GCN5. (**c**) CGNs transfected with siGCN5a and siGCN5b together with pCMV-EGFP plasmids were subjected to nucleic staining (scale bar=20 *μ*m), and the apoptotic rate was determined by scoring the percentage of GFP-positive neuron population with nuclear pyknosis. Mean±S.E.M., *n*=3, *P<0.05. GFP, green fluorescent protein

As pharmacological inhibitors might have off-targeted effects, we then introduced small interference RNAs (siRNAs) to specifically knockdown GCN5. The efficiency of the knockdown of two GCN5 siRNA fragments was first confirmed by abolishing the endogenous GCN5 expression in rat C6 glioma cells (Figure 2a). Furthermore, the siGCN5 fragments can efficiently silence GCN5 expression in CGNs (Figures 2b, *P*<0.05). As expected, the knockdown of GCN5 induced an obvious increase in apoptosis (Figures 2c, *P*<0.05). These results indicated that loss of GCN5 activity substantially induces neuronal apoptosis.

### Inhibition of GCN5 transcriptionally upregulates Bim

**Figure 3:**
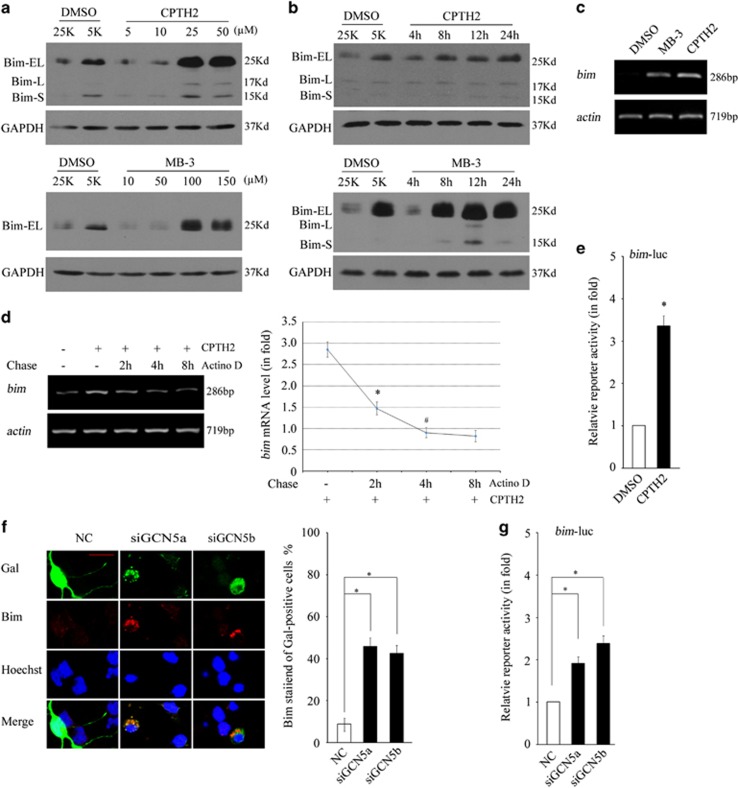
Inhibition of GCN5 transcriptionally upregulates Bim. (**a**) CGNs treated with CPTH2 and MB-3 in 25K media at the indicated doses for 24 h and Bim was detected by WB. 5K treatment causes a Bim induction as a positive control. GAPDH was reprobed to verify equal loadings. (**b**) CGNs treated with CPTH2 (50 *μ*M) and MB-3 (150 *μ*M) in 25K media for the indicated time course were subjected to WB to detect Bim expression. (**c**) CGNs treated with CPTH2 (50 *μ*M) and MB-3 (150 *μ*M) in 25K media for 12 h were subjected to RT-PCR to detect *bim* mRNA level. The *actin* was amplified to verify equal loadings. (**d**) Pulse chase assay: CGNs treated with CPTH2 (50 *μ*M) in 25K media for 18 h were subjected to further co-treatment of CPTH2 with Actinomycin D (2 *μ*M) for 2, 4 and 8 h, and then to RT-PCR. Changes in *bim* mRNA levels were determined as changes in band intensity after normalization to *actin* by the ImageJ software. (**e**) DIV5 CGNs transfected with bim-luc reporter for 12 h were administered with CPTH2 (50 *μ*M) in 25K media for 24 h and subjected to dual reporter analysis. (**f**) CGNs transfected with NC, siGCN5a and siGCN5b were subjected to immunofluorescence to detect Bim expression (scale bar=10 *μ*m). (**g**) CGNs transfected with bim-luc reporter together with NC, siGCN5a and siGCN5b for 48 h were subjected to dual reporter analysis. Mean±S.E.M., *n*=3, *P<0.05. DMSO, dimethyl sulphoxide; GAPDH, glyceraldehyde 3-phosphate dehydrogenase

Previous work has demonstrated that Bim upregulation is critical for apoptotic occurrence in CGNs.^29, 30, 31^ We then explored whether Bim is involved in the loss of GCN5-induced apoptosis. Indeed, both CPTH2 and MB-3 dose- and time course-dependently induced Bim expression. Bim induction started at 8 h posttreatment, earlier than the Caspase 3 activation and the increase in nuclear pyknosis. The *bim* mRNA levels were also upregulated following GCN5 inhibition (Figures 3a–c). A pulse chase assay showed that the increased *bim* mRNA levels predominantly came from new transcription but not mRNA stabilization (Figures 3d, *P*<0.05). Furthermore, the *bim* promoter activity monitored by a bim-luciferase reporter was upregulated following CPTH2 treatment or GCN5 knockdown (Figures 3e and g, *P*<0.05). The results suggest that loss of GCN5 transcriptionally upregulates Bim.

### Bim mediates apoptosis induced by inhibition of GCN5

**Figure 4:**
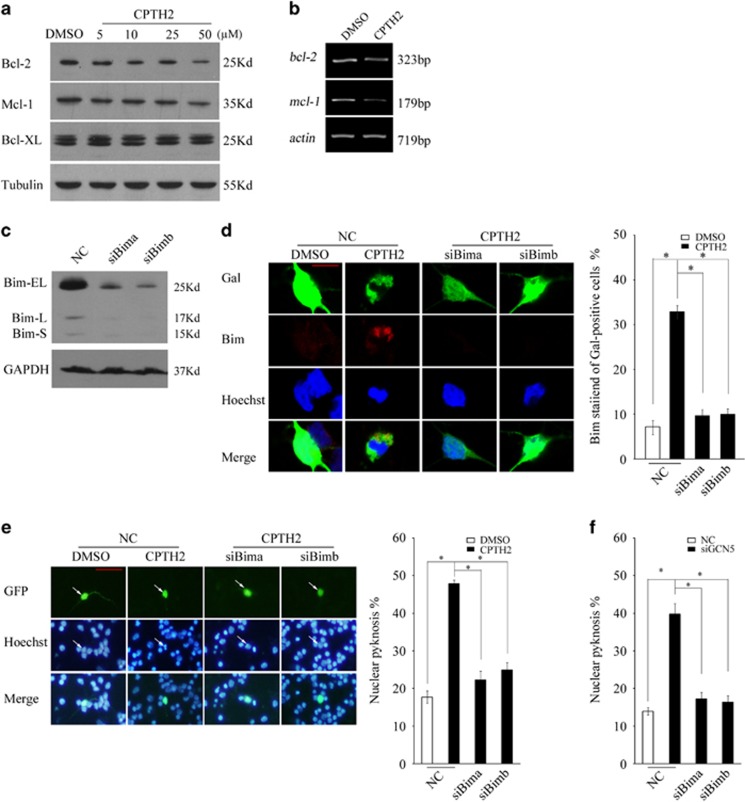
Knockdown of Bim rescues the loss of GCN5-induced apoptosis. (**a** and **b**) CGNs treated with CPTH2 at the indicated doses or 50 *μ*M in 25K for 24 h were subjected to WB or RT-PCR to detect Bcl-2, Mcl-1 or Bcl-XL expression. (**c**) Rat C6 glioma cells transfected with NC, siBima and siBimb were subjected to WB to detect Bim expression. (**d**) CGNs were transfected with NC, siBima and siBimb together with pCMV-Gal. Forty-eight hours later, neurons were exposed to CPTH2 (50 *μ*M) in 25K media for 24 h and subjected to immunofluorescence to detect Bim expression (scale bar=5 *μ*m). (**e**) CGNs were transfected with NC, siBima and siBimb together with pCMV-EGFP. Forty-eight hours later, neurons were exposed to CPTH2 (50 *μ*M) in 25K media for 24 h and subjected to nucleic staining (scale bar=20 *μ*m) and apoptotic analysis as in Figure 2c. White arrows indicate the transfected cells. (**f**) CGNs transfected with NC, siBima and siBimb together with siGCN5a and pCMV-EGFP plasmids were subjected to nucleic staining and apoptotic analysis as above. Mean±S.E.M., *n*=3, *P<0.05. DMSO, dimethyl sulphoxide; GAPDH, glyceraldehyde 3-phosphate dehydrogenase; GFP, green fluorescent protein

As Bim induction is prior to the activation of Caspase 3, it is reasonable to suppose that the induced Bim contributes to GCN5 loss-caused apoptosis. However, a previous study demonstrated that the induced Bim is primarily sequestered by Bcl-2 family members Bcl-2, Bcl-XL and Mcl-1,^32^ which would neutralize its pro-apoptotic roles. We then determined whether GCN5 inhibition changes these three proteins or their association with Bim. Excitingly, CPTH2 treatment dose-dependently suppressed Bcl-2 and Mcl-1 expression but without affecting Bcl-XL expression. Furthermore, bcl-2 and mcl-1 mRNA levels downregulate following GCN5 inhibition (Figures 4a and b). The results support that GCN5 inhibition-mediated suppression on the two proteins might facilitate Bim to promote apoptosis. Indeed, the knockdown of Bim completely abrogated the apoptosis induced by CPTH2 as well as the silencing of GCN5 (Figures 4c–f, *P*<0.05). The results indicate that inhibition of GCN5 leads to Bim-dependent apoptosis.

### GCN5 inhibition-activated Egr-1 and E2F1 contributes to Bim induction

**Figure 5:**
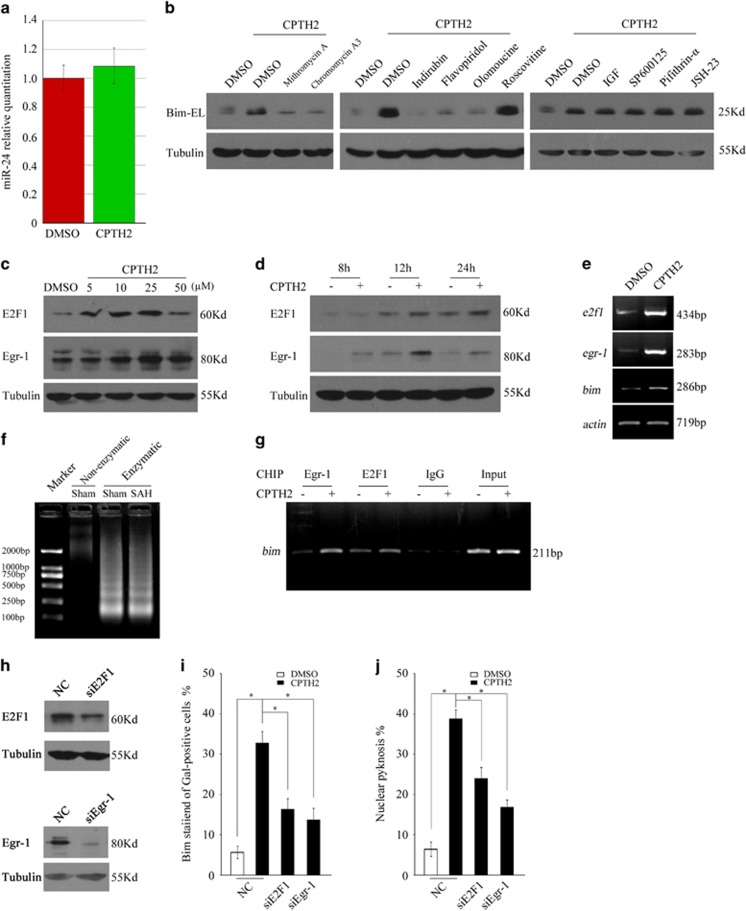
GCN5 inhibition induces Bim expression through transactivating Egr-1 and E2F1. (**a**) CGNs were exposed to CPTH2 or dimethyl sulphoxide (DMSO) in 25K media for 18 h, small mRNAs were extracted and reverse transcribed as templates for Q-PCR to detect miR-24 expression and U6 small nuclear RNA (snRNA) was amplified to normalize the miR24 outputs. (**b**) CGNs in 25K media exposed to CPTH2 alone or together with indirubin-3'-oxime (10 *μ*M), flavopiridol (2 *μ*M), mithramycin A (10 *μ*M), chromomycin A3 (0.3 *μ*M), olomoucine (200 *μ*M), roscovitine (50 *μ*M), IGF (100 ng/ml), SP600125 (10 *μ*M), pifithrin-alpha (2 *μ*M) or JSH-23 (5 *μ*M) for 24 h were subjected to WB to detect Bim expression; tubulin was reprobed to verify equal loadings. (**c**–**e**) CGNs in 25K media treated with CPTH2 at the indicated doses for 24 h or treated with CPTH2 (50 *μ*M) for the indicated time courses were subjected to WB or RT-PCR to detect E2F1 and Egr-1 expression in protein or mRNA. (**f** and **g**) The cleaved chromatin fragments were immunoprecipitated by anti-E2F1 or Egr-1 antibody and then purified as templates to amplify the region of bim promoter by PCR. The part of chromatin fragments before IP was purified as templates to amplify bim promoter as a reference. (**h**) Rat C6 glioma cells transfected with NC, siE2F1 and siEgr-1 were subjected to WB to detect the E2F1 or Egr-1 expression. Tubulin was reprobed to verify equal loadings. (**i**) DIV5 CGNs transfected with NC, siE2F1 and siEgr-1 together with pCMV-Gal plasmids for 36 h were exposed to CPTH2 (50 *μ*M) in 25K media for 18 h and then were subjected to immunofluorescence to detect the Bim expression. The percentage of stained *β*-Gal-positive neuron population with Bim was calculated. (**j**) DIV5 CGNs transfected with NC, siE2F1 and siEgr-1 together with p-GFP plasmids for 36 h were exposed to CPTH2 (50 *μ*M) in 25K media for 24 h and then were subjected to nucleic staining and apoptotic analysis as in Figure 2c. Mean±S.E.M., *n*=3, *P<0.05

To explore the mechanisms that are involved in Bim induction following GCN5 inhibition, we first examined whether CPTH2 treatment can repress the expression of miR-24, a microRNA which is known to negatively regulate Bim expression.^33^ Q-PCR results showed that miR-24 levels in the CPTH2 group are not significantly different from those in control (Figure 5a, 0.05<*P*>0.05), indicating that Bim induction following GCN5 inhibition is not through alteration of miR-24 expression.

Next, we attempted to identify the potential transcriptional factors that could be implicated in Bim upregulation, including E2F1, FOXO1/3a, Egr-1, p53 and NF-*κ*B, which have been demonstrated to directly target the Bim gene.^29, 30, 34, 35, 36^ Interestingly, inhibition of both the Egr-1 transcriptional activity by mithramycin A and chromomycin A3 and the CDK activities by the pan-CDK inhibitors indirubin-3'-oxime and flavopiridol remarkably blocked the CPTH2-induced Bim expression (Figure 5b). Furthermore, administration of olomoucine, a specific inhibitor for CDK4/6, but not roscovitine, a specific inhibitor for CDK2/5, abrogated the Bim induction (Figure 5b), suggesting that CDK4/6 activation contributes to Bim regulation following GCN5 inhibition. However, inhibition of Forkhead activity by IGF, JNK/c-Jun activity by SP600125, p53 activity by pifithrin-alpha or NF-kB activity by JSH-23 do not rescue the CPTH2-evoked Bim expression (Figure 5b).

To further determine that Egr-1 and CDK4/6 activation contribute to the Bim induction, we tested whether Egr-1 and E2F1, a critical transcriptional factor downstream of CDK4/6,^37^ are activated following GCN5 inhibition. As shown in Figures 5c–e, both Egr-1 and E2F1 were induced in a time- and dose-dependent manner by CPTH2. Consistently, *egr-1* and *e2f1* mRNA levels were also upregulated. Chromatin immunoprecipitation (ChIP) assay showed that the induced E2F1 and Egr-1 bind to the region (−2554 to −2343 bp) of *bim* promoter (Figures 5f and g), distinct from the originally identified region (−221 to +106 bp) (for details, see Discussion section and Supplementary Figure S5). Furthermore, knockdown of E2F1 or Egr-1 expression by siRNAs effectively rescued CPTH2-caused Bim induction, as well as apoptosis (Figures 5h–j, *P*<0.05). Together, the results clearly indicate that the GCN5 inhibition-activated E2F1 and Egr-1 contributes to Bim induction.

### Potassium deprivation or glutamate exposure causes a loss of GCN5 activity, in contrast to E2F1, Egr-1 and Bim induction

**Figure 6:**
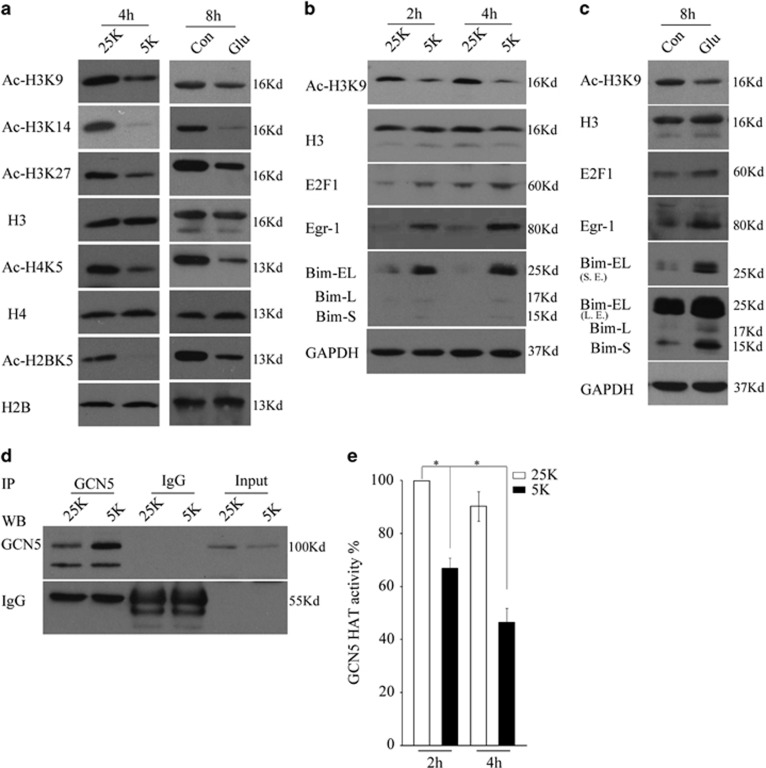
GCN5 loses its activity following potassium deprivation or glutamate exposure. (**a**) DIV 7 CGNs treated with 25K or 5K for 4 h, or glutamate (Glu, 100 *μ*M), were subjected to WB to detect Ac-H3K9, Ac-H3K14, Ac-H3K27, H3, Ac-H4K5, H4, Ac-H2BK5 and H2B. Con: control. (**b**) CGNs treated with 25K and 5K for two time durations 2 and 4 h were subjected to WB to detect Ac-H3K9, H3, E2F1, Egr-1 and Bim. (**c**) CGNs exposed to glutamate in 25K media for 8 h were subjected to WB to detect Ac-H3K9, H3, E2F1, Egr-1 and Bim. (**d** and **e**) CGNs treated with 25K and 5K for 4 h were subjected to IP with anti-GCN5 antibody and then to WB to detect the precipitated GCN5 (partial) and the left precipitated complexes were subjected to *in vitro* HAT activity. Mean±S.E.M., *n*=3, *P<0.05. GAPDH, glyceraldehyde 3-phosphate dehydrogenase

CGNs undergo a typical apoptosis following potassium deprivation or glutamate, and a transcriptional upregulation of Bim has been implicated in the processes.^29, 38^ We then asked whether the loss of GCN5 activity is involved in potassium deprivation- or glutamate-induced Bim upregulation and apoptosis. Greatly,CGNs treated with 25K or 5K for 4 h or glutamate (in 25K) for 8 h caused a dramatic reduction in the acetylation of H3K9, meaning that GCN5 activity decreases (Figures 6a and 1a). Contrary to this, Bim, as well as E2F1 and Egr-1, are robustly induced (Figures 6b and c). Furthermore, 5K or glutamate treatment also results in a reduction in acetylation of other histone lysine sites, including H2BK5, H3K14, H3K27 and H4K5, suggesting that a loss of global acetylation occurs.

As a further test, *in vitro* HAT activity assays showed that 5K treatment for 2 h causes a significant decrease in GCN5 activity by 35% compared with control 25K and, for 4 h, a greater decrease by 58% (*P*<0.05). Interestingly, GCN5 also loses its binding capacity to Bim promoter following potassium deprivation (Supplementary Figures S2a and b). Together, the results suggest that potassium deprivation leads to a substantial reduction in GCN5 activity, contrary to the E2F1, Egr-1 and Bim induction.

### Overexpression of GCN5 suppresses Egr-1, E2F1 and Bim expression and apoptosis

**Figure 7:**
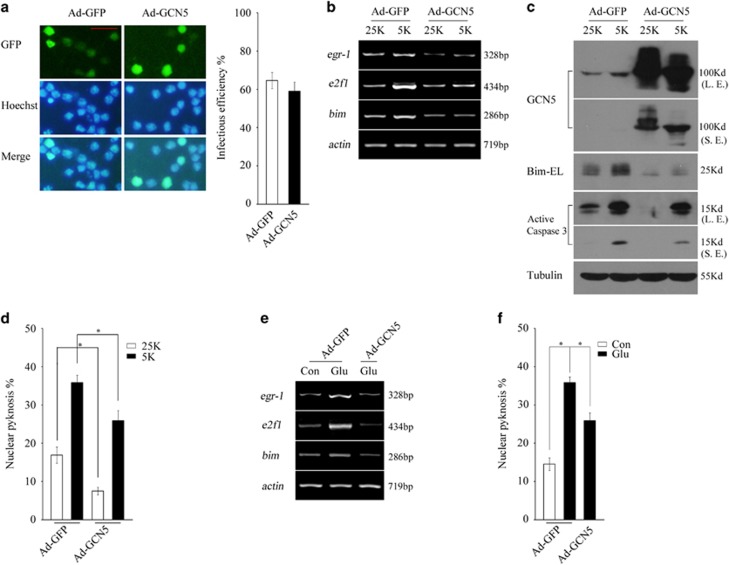
Overexpression of GCN5 suppresses Bim expression and apoptosis following potassium deprivation or glutamate exposure. (**a**) DIV 5 CGNs were infected with Ad-GFP or Ad-GCN5 at 100 multiplicity of infection. After 48 h, the infected neurons were subjected to nucleic staining (scale bar=20 *μ*m) to determine infectious efficiency or RT-PCR to determine *e2f1*, *egr-1* and *bim* mRNA level. (**b** and **c**) Forty-eight hours posttransfection, the neurons infected as in panel (**a**) were subjected to 25K and 5K treatment for 6 h and then to RT-PCR to detect *e2f1*, *egr-1*, and *bim* mRNA level, and WB was performed to detect GCN5, Bim and Caspase 3. L.E.: long time of exposure; S.E.: short time of exposure. (**d**) Forty-eight hours posttransfection, the neurons infected as in panel (**a**) were subjected to 25K and 5K treatment for 12 h and the apoptotic rate was determined as in Figures 1b. (**e** and **f**) Forty-eight hours posttransfection, the neurons infected as in panel (**a**) were exposed to glutamate (Glu, 100 *μ*M) in 25K media for 12 h followed by RT-PCR to detect *e2f1*, *egr-1* and *bim* mRNA level or for 24 h and the apoptotic rate was determined as above. Mean±S.E.M., *n*=3, *P<0.05. GFP, green fluorescent protein

As potassium deprivation or glutamate-evoked Bim induction and apoptosis are accompanied by a loss of GCN5 activity, we then assessed whether elevating GCN5 activity can rescue the processes by introducing adenovirus-mediated overexpression of GCN5 in CGNs. Ad-GCN5 or the control Ad-GFP showed a high infectious efficiency in neurons, up to >60% (Figure 7a). Indeed, overexpression of GCN5 not only greatly suppressed basal Bim expression in the 25K condition but also remarkably inhibits 5K or glutamate-induced E2F1, Egr-1 and Bim expression. Accordingly, overexpression of GCN5 significantly reduces Caspase 3 activity and the apoptotic rate in 25K, 5K or glutamate conditions (Figures 7b–f, *P*<0.05). The results suggest that GCN5 has important roles in negatively modulating Bim expression and neuronal apoptosis.

### SAH results in a transcriptional upregulation of Bim, E2F1 and Egr-1 but a downregulation of Bcl-2 and a loss of GCN5 activity

Recently, neuronal apoptosis was found to be implicated in the EBI following SAH, featured with a robust Bim induction.^39, 40, 41^ We then asked whether the events above demonstrated *in vitro* are involved in SAH-caused Bim induction and apoptosis in rats, including a loss of GCN5 activity, a transcriptional upregulation of E2F1 and Egr-1 and a downregulation of Bcl-2 and Mcl-1.

**Figure 8:**
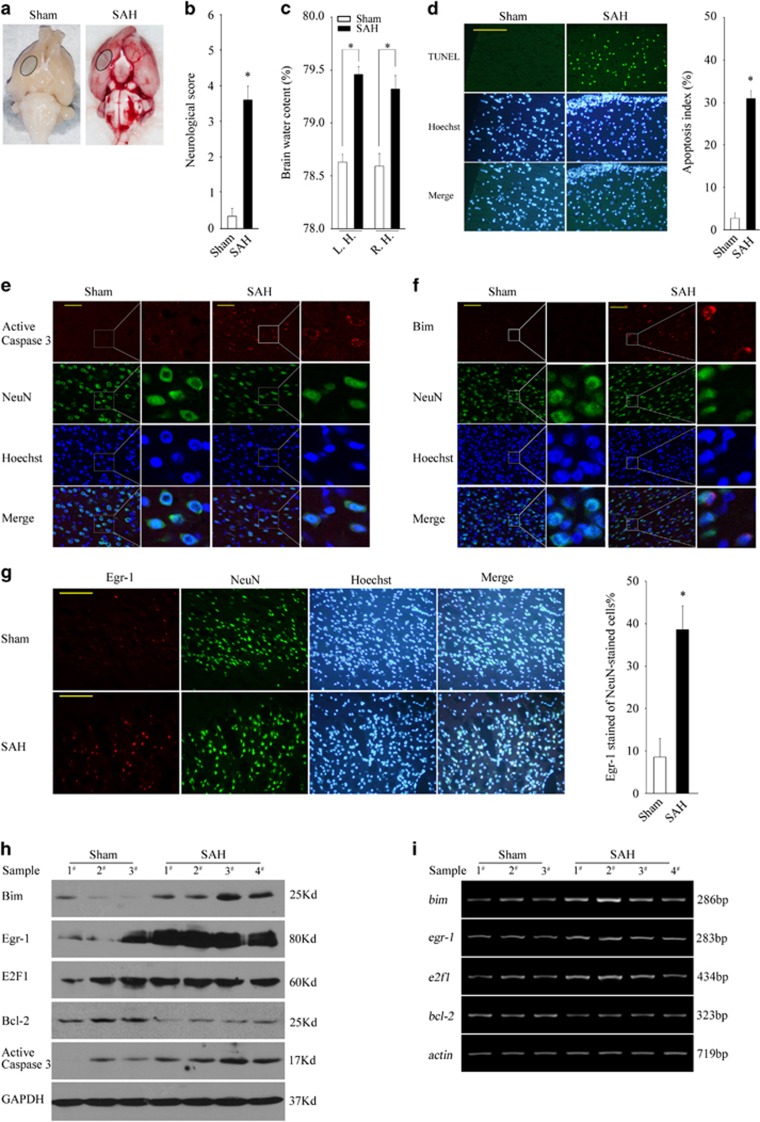
SAH results in a typical neuronal apoptosis, concomitant with a transcriptional upregulation of Bim, E2F1 and Egr-1 but a downregulation of Bcl-2. (**a**) Representative pictures of the brains are shown after surgery. In SAH rat, significant blood clots mainly distribute at the base of Willis circle and brain stem. The same part of basal cortical brain tissue was taken for tests (circled areas). (**b** and **c**) At 24 h after SAH, neurobehavioral performance or brain water content in the left or right hemisphere (L.H or R.H.) was tested. (**d**) TUNEL (terminal deoxinucleotidyl transferase-mediated dUTP-fluorescein nick end labelling) assay was performed to test the apoptosis rate at 24 h after SAH, scale bar=50 *μ*m. (**e**–**g**) The active caspase 3, Bim and Egr-1 were detected by immunofluorescence at 24 h after SAH and their positive rates of all NeuN-stained cells were calculated, scale bar in panel (**e**)=20 *μ*m and in panels (**f** and **g**)=50 *μ*m. (**h** and **i**) The protein expression levels or mRNA levels of Bim, Egr-1, E2F1 and Bcl-2 were determined by WB or RT-PCR at 24 h after SAH. The values are expressed as mean±S.E.M., *P<0.05, *n*=6 in each group. GAPDH, glyceraldehyde 3-phosphate dehydrogenase

At 24 h after SAH, the animals in the SAH group demonstrated severe neurobehavioral dysfunction and markedly increased brain water content in both the left and right hemispheres compared with the sham group (Figures 8b and c, *P*<0.05). By taking the same part of basal cortical brain tissue marked in Figure 8a for further tests, we found that the rate of TUNEL-positive cells in the SAH group was much higher than that in the sham group. Similarly, the rate of nuclear irregularity in SAH also significantly increased (Figure 8d and Supplementary Figure S3a, *P*<0.05). Furthermore, the active Caspase 3 mainly occurred in cells co-stained with NeuN in the SAH group but barely in the sham group (Figure 8e and Supplementary Figures S3b and c, *P*<0.05), suggesting that SAH leads to a typical neuronal apoptosis.

Accompanied with the apoptosis, Bim and Egr-1 expression remarkably increased in NeuN-positive neurons but not in GFAP-positive glia in the SAH group (Figures 8f, g and Supplementary Figure S3d, *P*<0.05). Furthermore, SAH caused a transcriptional upregulation of Bim, Egr-1 and E2F1 and a downregulation of Bcl-2 (Figures 8h, i, Supplementary Figures S3e and f, *P*<0.05) but without changing Mcl-1 expression (data not shown).

**Figure 9:**
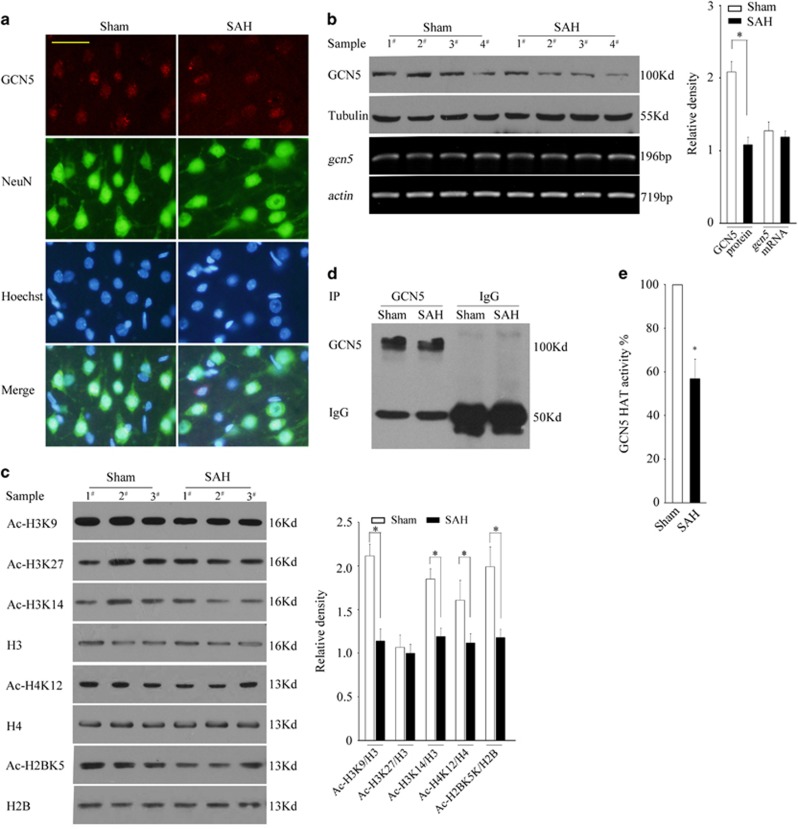
GCN5 loses its activity following SAH. (**a**) The location of GCN5 in the neurons of sham or SAH rat were determined by co-staining with GCN5 and NeuN, scale bar=20 *μ*m. (**b**) The protein expression levels or mRNA levels of GCN5 were determined by WB or RT-PCR at 24 h after SAH. (**c**) At 24 h after SAH, the homogenized brain tissues were subjected to a histone extraction followed by WB to detect Ac-H3K9, Ac-H3K14, Ac-H3K27, H3, Ac-H4K12, H4, Ac-H2BK5 and H2B. (**d** and **e**) At 24 h after SAH, the homogenized brain tissues were subjected to IP and the partial precipitated complexes were subjected to WB and the left ones were subjected to *in vitro* HAT activity assay. The values are expressed as mean±S.E.M., *P<0.05, *n*=6 in each group

**Figure 10:**
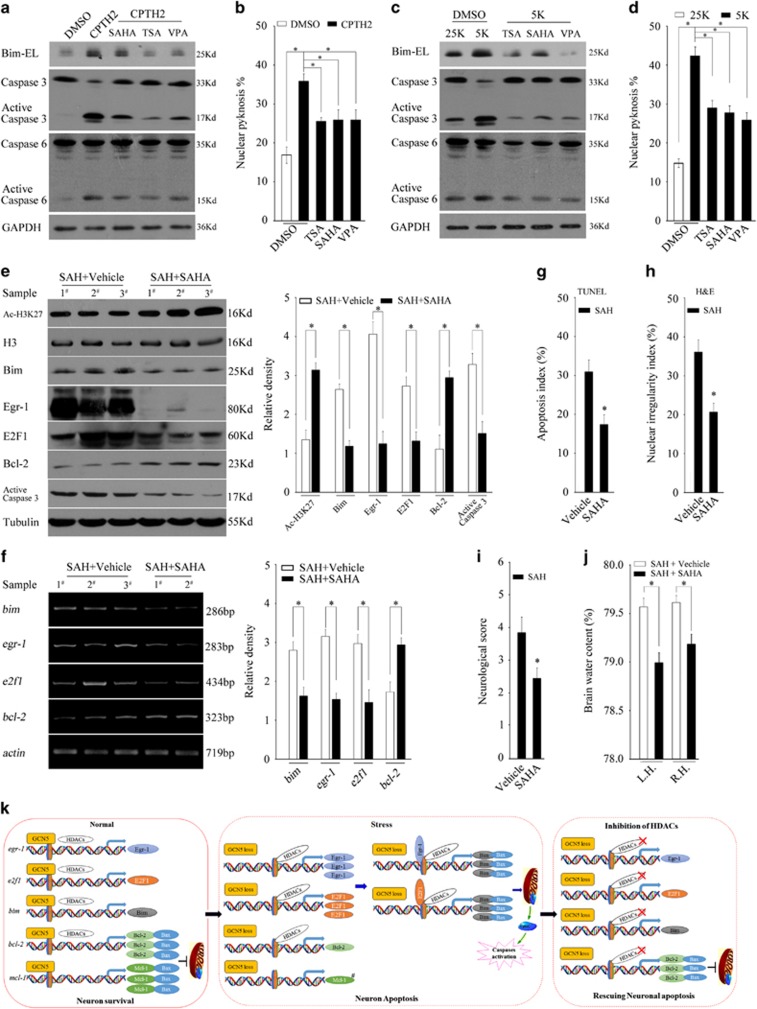
pan-HDAC inhibitors suppress Bim expression and apoptosis induced by loss of GCN5, potassium deprivation or SAH. (**a** and **b**) CGNs in 25K media treated with DMSO, CPTH2 (50 *μ*M) or CPTH2 together with SAHA (1 *μ*M), TSA (0.5 *μ*M) and VPA (6 mM) for 24 h were subjected to WB to detect Bim and Caspase 3, Caspase 6 or subjected to nucleic staining to determine the apoptotic rates as in Figure 1b. (**c** and **d**) CGNs treated with 25K, 5K or 5K together with SAHA (1 *μ*M), TSA (0.5 *μ*M) and VPA (6 mM) for 12 h were subjected to WB to detect Bim and Caspase 3 or subjected to nucleic staining to determine the apoptotic rates. (**e** and **f**) The mRNA levels or protein expression levels of Bim, Egr-1, E2F1 and Bcl-2 were determined by RT-PCR or WB at 24 h after SAH in SAH+vehicle rats or SAH+SAHA ones. The activity of Caspase 3 was also determined. (**g** and **h**) At 24 h after SAH, TUNEL (terminal deoxinucleotidyl transferase-mediated dUTP-fluorescein nick end labelling) or haematoxylin nd eosin (H&E) staining was performed to compare the difference of the apoptotic rate or the nuclear irregularity rate between the SAH+vehicle and SAH+SAHA groups. (**i** and **j**) At 24 h after SAH, the difference of neurobehavioral performance or brain water content in the left or right hemisphere (L.H or R.H.) was compared between the SAH+vehicle and SAH+SAHA groups. The values are expressed as mean±S.E.M., *P<0.05, *n*=6 in each group. (**k**) A schematic diagram illustrating the possible mechanisms involved in neuronal apoptosis following loss of GCN5 activity. In normal conditions, the activity of GCN5 is implicated in transcriptionally repressing Egr-1, E2F1 and Bim but promoting Bcl-2, and Mcl-1 expression to prevent Bax (or Bak) from permeabilizing the mitochondria for survival. When neurons are subjected to stress such as potassium deprivation or SAH, GCN5 loses its HAT activity, which leads to a HDAC activity-dependent upregulation of Egr-1 and E2F1 but a downregulation of Bcl-2 and Mcl-1(#no significant change between sham and SAH rats). The active Egr-1 or E2F1 transcriptionally upregulates Bim to activate Bax to increase the permeability of mitochondria for Cytochrome *C* and the reduction of Bcl-2 or Mcl-1 frees Bim proteins to facilitate the process. As a result, the Caspases are activated and apoptosis occurs. Inhibition of HDACs significantly rescues the stress-evoked events and neuronal apoptosis. DMSO, dimethyl sulphoxide; GAPDH, glyceraldehyde 3-phosphate dehydrogenase

Next, we examined whether SAH treatment caused a change in GCN5 activity. As shown in Figure 9a, GCN5 was mainly expressed in NeuN-positive neurons. Interestingly, SAH caused a significant decrease in GCN5 expression but without affecting the *gcn5* mRNA levels (Figure 9b, *P*<0.05). As a result, the acetylation of H3K9 and GCN5 HAT activity remarkably reduced in the SAH group (Figures 9c–e, *P*<0.05). Furthermore, SAH also caused a decrease in Ac-H3K14, Ac-H4K12 and Ac-H2BK5 but without affecting Ac-H3K27 (Figure 10c, *P*<0.05), suggesting that global acetylation homoestasis during SAH-induced EBI is impaired.

Together, SAH results in a loss of GCN5 activity, concomitant with a transcriptional upregulation of Egr-1, E2F1 and Bim but a downregulation of Bcl-2.

### Inhibition of HDACs rescues Bim induction and apoptosis following GCN5 inhibition, potassium deprivation or SAH

As loss of acetylation often inclines to the function of HDACs, we then determined whether Bim upregulation and neuronal apoptosis can be rescued by inhibition of HDACs. Indeed, three pan-HDAC inhibitors, including TSA, SAHA and VPA, can remarkably suppress Bim expression, as well as Egr-1 and E2F1, induced by either exposure of CPTH2 or potassium deprivation in CGNs. Paralleling with these, the activity of Caspase 3 and 6 reduced accordingly (Figures 10a, c, Supplementary Figures S4a and b). Consistently, HDAC inhibition significantly decreased the apoptotic rates evoked by CPTH2 or potassium deprivation compared with the respective control (Figures 10b and d, *P*<0.05). The results suggest that inhibition of HDAC can effectively rescue Bim-dependent CGN apoptosis under loss of GCN5 activity.

Next, we determined whether inhibition of HDAC by SAHA can rescue SAH-induced upregulation of Egr-1, E2F1 and Bim and significantly ameliorate EBI. As shown in Figure 10e, administration of SAHA causes a remarkable increase in Ac-H3K27, suggesting that the activity of HDACs was efficiently blocked. In SAH rats, SAHA administration led to a marked decrease in the Egr-1, E2F1 and Bim, as well as their mRNA levels, compared with the vehicle injection, but an increase in Bcl-2 protein and mRNA (Figures 10e and f). Consistent with these changes, SAH-induced apoptosis was significantly alleviated by SAHA (Figures 10f–h, *P*<0.05). Consequently, administration of SAHA led to a substantial improvement in the neurological score (Figures 10i, *P*<0.05) and alleviated brain oedema in SAH rats compared with vehicle (Figure 10j, *P*<0.05). Together, inhibition of HDAC significantly rescues Bim induction and apoptosis following loss of GCN5 activity *in vitro* and *in vivo*.

## Discussion

A fact has been highlighted that several neurodegenerative or brain injury conditions can be ameliorated by treatment with various HDAC inhibitors. Oxidative stress, reported to be closely associated with these diseases, fails to induce neuronal apoptosis when cells are treated with HDAC inhibitors.^42^ These strongly point towards a loss of neuronal acetylation homeostasis during neuronal apoptosis and HDAC members are implicated in the process. However, among the 18 HDAC members, the role for each individual HDAC in regulating neuronal survival or death remains quite different. For example, the activity of HDAC1, 4, 5 or 6 contributes to neuronal apoptosis,^43, 44, 45, 46, 47^ whereas HDAC2, 3, 7 or Sirt1 are known to promote neuronal survival.^9, 38, 48, 49, 50^ The current evidence cannot completely explain the antiapoptotic effects of HDACIs in neurons because a global inhibition of HDACs by the pan-HDACIs would result in compromised effects between pro-survival HDAC signals and pro-apoptotic ones. Therefore, the critical event of acetylation loss-coupled neuronal apoptosis needs further exploration.

Notably, loss of basal HAT activity alters the precious HAT:HDAC balance towards enhanced histone deacetylation, resulting in a loss of acetylation homeostasis. Thus the effect of a loss of HAT activity on neuronal apoptosis deserves attention. In fact, previous studies have investigated the functions of HAT members in modulating neuron fate, and yet increasing HAT activities are often associated with apoptosis. For example, enhancing p300 HAT activity by upregulating PKC*δ* expression promotes the apoptosis of dopaminergic neurons.^51^ Similarly, Tip60 HAT activity mediates APP-induced lethality and apoptosis in neurons in the AD model.^52^ Furthermore, CBP/p300 cooperating with NF-Y and FOXO3a triggers apoptosis in sympathetic neurons.^53^ CBP promotes apoptosis in normal CGNs but loss of CBP by Caspase 6 facilitates neuronal apoptosis following potassium deprivation,^54^ suggesting that CBP exhibits different roles in regulating neuronal fate in different conditions.

In this study, we found that inhibition of GCN5 by CPTH2, a synthesized molecule derived from thiazole in budding yeast,^28^ or by GCN5 siRNAs is sufficient to induce neuronal apoptosis. Whereas inhibition of p300/CBP or Tip60 by C646 and MG-149, two small molecules that specifically inhibit the HAT activity by docking into the Lys-CoA-binding pocket,^55, 56^ respectively, does not lead to an obvious apoptosis, suggesting that although selective regulation of acetylation of promoters results in differential control of gene transcription, whether a loss of HAT:HDAC balance is associated with neuronal death largely depends on whether the protein belongs to proapoptotic or antiapoptotic groups of proteins. Indeed, the critical evidence for the loss of GCN5-coupled neuronal apoptosis is the transcriptional upregulation of Bim, accompanied by a downregulation of Bcl-2 and Mcl-1. The reduction of Bcl-2 and Mcl-1 frees Bim proteins to activate Bax or Bak and subsequently increases the mitochondria permeability, facilitating Caspase-dependent apoptosis.^32^ These results strongly suggest that the loss of GCN5 activity is a critical event among the epigenetic changes that trigger neuronal apoptosis.

However, in our CHIP assays shown in Figure 5g, we found that the induced Egr-1 and E2F1 binds to the region spanning −2554 to −2344 bp of the *bim* promoter following GCN5 inhibition, which is distinct from the region (−221 to +106 bp) originally identified to be regulated by the two transcriptional factors.^29, 34, 57^ By using other primer pairs to amplify the adjacent parts of the region (−2554 to −2344 bp), we confirmed that Egr-1 binds to a large range spanning −3022 to −2233 bp (~789 bp), whereas E2F1 binds to a region spanning −2554 to −2233 bp (~321 bp). Interestingly, no E2F1 was detected binding to the region (−221 to +106 bp), consistent with the previous report,^34^ although the induced Egr-1 binding to the region was detectable following GCN5 inhibition but very faint compared with that to the region (−3022 to −2233 bp) (Supplementary Figures S5a and b). Bioinformatics analysis indicates that three putative Egr-1 response sites (named as Egr-1-like site-1, 2, 3), which have been implicated in Egr-1-mediated regulation of Bim or Noxa transcription,^58, 59^ locate at the Egr-1 binding region, and one putative E2F site (TTGGGGC, E2F-like site) exists in the ~321 bp region,^60^ suggesting that the range spanning −3022 to −2233 bp could be a new critical region regulated by GCN5, Egr-1 and E2F1.

In conclusion, we demonstrated here that GCN5 activity is essential for survival and the loss of GCN5 activity results in a typical apoptosis in matured neurons. Mechanistically, in normal homeostasis, GCN5 activity is implicated in transcriptionally repressing Egr-1, E2F1 and Bim but promoting Bcl-2, Mcl-1 expression to prevent Bax or Bak from permeabilizing the mitochondria for survival. In neurons wherein global acetylation is impaired following potassium deprivation, glutamate or SAH, the loss of GCN5 activity-evoked E2F1- and Egr-1-dependent transcriptional upregulation of Bim is a critical step in triggering apoptosis, the concomitant downregulation of Bcl-2 and Mcl-1 might facilitate the process.^32^ Promisingly, HDAC inhibitors can significantly rescue the loss of GCN5-caused Bim induction and apoptosis, and two FDA-approved drugs, VPA and SAHA, are in hand (a schematic diagram illustrating the possible mechanisms is shown in Figure 10k).

## Materials and methods

### Neuronal culture and potassium deprivation

Rat CGNs were prepared from 7–8-day old Sprague-Dawley rat pups (15–19 g) as described previously.^28^ Briefly, neurons were dissociated from freshly dissected cerebella by mechanical disruption in the presence of trypsin and DNase and then seeded at a density of 1.5 × 10^6^ cells/ml in basal modified Eagle's medium containing 10% foetal bovine serum and 25 mM KCl (25K+S). Cytosine arabinoside (10 *μ*M) was added 24 h after seeding to limit the growth of non-neuronal cells. For potassium deprivation, experiments were performed as described previously.^28^ Briefly, cells cultured *in vitro* for 7 days (DIV7) in medium containing 25K+S were switched into serum-free medium containing 25 or 5 mM KCl (25K or 5K) in the presence or absence of the inhibitors CPTH2, MB-3, C646, MG-149, SAHA, VPA and TSA, indirubin-3'-oxime, flavopiridol, mithramycin A, chromomycin A3, olomoucine (200 *μ*M), roscovitine, IGF, SP600125, pifithrin-alpha or JSH-23 (Sigma, Shanghai, China). Cells that did not receive inhibitors received DMSO as a control. The final concentration of DMSO was <0.2%. For glutamate exposure, neurons were switched into 25K+glutamate (100 *μ*M). The glutamate stock solution is 1000 × 100 *μ*M in 1 N HCl.

### Western blotting (WB)

WB analysis was performed as described in detail previously.^28, 61^ For tissue analysis, rats were killed at 24 h after SAH induction, and the cortical samples were homogenized in RIPA buffer (Sangon, Shanghai, China) and centrifuged at 12 000 × *g* for 15 min at 4 °C. The supernatants were collected and protein concentrations were determined with a BCA Kit (Thermo Fisher Scientific, Rockford, IL, USA). Equal amounts (60 *μ*g) of protein per sample were subjected to WB. The antibodies used include: GCN5 (CST, no. 3305), H2B (CST, no. 2722), H3 (CST, no. 9715), H4 (CST, no. 2592), Ac-H2BK5 (CST, no. 2574), Ac-H3K9 (CST, no. 9671), Ac-H3K14 (CST, no. 7627), Ac-H3K27 (CST, no. 4353), Ac-H4K12 (CST, no. 2591), Ac-H4K5 (CST, no. 8647), Caspase 3 (CST, no. 9662), Caspase 6 (CST, no. 9762), Bim (CST, no. 3305), E2F1 (CST, no. 3742), Egr-1 (CST, no. 4154), GAPDH (no. 2118) (Cell Signaling Technology; diluted at 1:1000), Flag, or tubulin (Sigma; both diluted at 1:10 000). The horseradish peroxidase-conjugated secondary antibodies are used (Jackson ImmunoRes, West Grove, PA, USA), and signals are visualized by using the ECL chemiluminescence system (Thermo Fisher Scientific, Rockford, IL, USA).

### Adenovirus infection

The recombinant adenovirus plasmid AdEasy Flag GCN5 got from Addgene (plasmid no. 14106, Cambridge, MA, USA) was originally constructed by Dr Pere Puigserver's laboratory.^62^ After digesting with PacI, the big fragments of constructs were transfected into 293A cells for packing and the positive colony efficiently expressing GCN5 was selected for further amplification and purification as described previously.^28^ Ad-GFP was amplified and purified as a control.

DIV5 CGNs were infected with Ad-GFP or Ad-GCN5 at a multiplicity of infection of 100 for 48 h and subjected to WB and reverse transcription-PCR (RT-PCR) for further analysis. Cells were stained with the DNA dye bisbenzimide (Hoechst 33258; 5 *μ*g/ml) to determine the infectious efficiency of the two adenovirus by scoring the rate of GFP-positive neurons in all cells in the same visual field.

### DNA fragmentation analysis (DNA ladder)

Cells were lysed in TE lysate buffer (0.2% Triton X-100, 50 mM Tris, 10 mM EDTA), and 500 *μ*l supernatants were added with 110 *μ*l 300 mM sodium acetate and 500 *μ*l isopropyl alcohol for extraction of DNA at −20 °C overnight. The precipitated DNA was resolved in 20 *μ*l 10 mM Tris (pH 8.5) containing RNAse A and 5 *μ*l DNA was analysed using gel electrophoresis (2% agarose). Photos were taken in a Gel Documentation System (WD-9413B, LIUYI, Inc, Beijing, China).

### ChIP assays

ChIP assays were performed using the Enzymatic ChIP Assay Kit (Cell Signaling Technology) according to the manual and as previously described.^28^ Two micrograms of rabbit E2F1 and Egr-1 antibody (Cell Signaling Technology) were used for immunoprecipitation (IP). Purified DNA was subjected to PCR amplification using the primers spanning the E2F1- and Egr-1-binding sites on the *bim* promoter (forward, 5′-TGCCACCAAAGATCTCTACC-3′ reverse, 5′-GCATTTCCTCACAGAGTTGG-3′.

### Reverse transcription-PCR

Total RNA was extracted and isolated from CGNs or brain tissue using the TRIzol reagent (Invitrogen, Carlsbad, CA, USA) as described previously.^28^ With the Primer Premier 5.0 software (PREMIER Biosoft, Palo Alto, CA, USA), we designed primers that were specific for *bim* (forward 5′-CTACCAGATCCCCACTTTTC-3′, reverse 5′-GCCCTCCTCGTGTAAGTCTC-3′); *e2f1* (forward 5′-GACTGTGACTTTGGGGACT-3′, reverse 5′-TGTTCACCTTCATTCCC-3′); *egr-1* (forward 5′-CGAGCGAACAACCCTACGAGC-3′, reverse 5′-GAGGCAGAGGAAGACGATGAAGC-3′); *bcl-2* (forward 5′-GGGATGCCTTTGTGGAACTA-3′, reverse 5'-ATTTGACCATTTGCCTGAAT-3'); *mcl-1* (forward 5'-GAAACAAAGAGGCTGGGATG-3', reverse 5'-GTAGTTGGTGGCTGGAGGTT-3')*, gcn5* (forward 5'-CATCGGTGGGATTTGCTT-3', reverse 5'-GTACTCGTCGGCGTAGGTG-3'); and *actin* (forward 5′-CAACTGGGACGATATGGAGAAG-3′ and reverse 5′-TCTCCTTCTGCATCCTGTCAG-3′).

### Quantitative real-time PCR and microRNA

Small RNA was extracted by RNAiso for Small RNA following the protocol from the producer (TAKARA, Dalian, China). The miRNA RT was conducted using the Hairpin-it microRNA and U6 snRNA Normalization RT-PCR Quantitation Kit (GeneParma, Shanghai, China). Optimized primers for amplifying miRNA-24 (miR-24) were used per the manufacturer's protocols. The qPCR was performed in ABI 7300 Real-Time PCR System (Applied Biosystems, Thermo Fisher Scientific, Waltham, MA, USA) and relative miR-24 expression was calculated using the formula ratio=2^−ΔΔCt^.

### Immunofluorescence

Immunocytochemistry and confocal imaging were performed as described previously.^28, 61^ For tissue analysis, the rats were subjected to perfusion–fixation 24 h after sham or SAH-induction surgery, and the frozen brain samples were cut into 20 *μ*m sections. One slice from every 6 serial cuttings in each segment was selected, and altogether 4–6 slices were collected for immunofluorescence. The slices were blocked with 5% normal donkey serum in PBS containing 0.3% Triton X-100 for 1 h at room temperature prior to incubation with primary antibody overnight at 4 °C. Secondary antibodies (FITC or CY3, 1:200) were incubated for 1 h at room temperature. After nucleic staining with Hoechst 33258, photos were taken on confocal (ZEISS, LSM 880, Göttingen, Germany) or fluorescence-inverted microscope (IX-71, Olympus, Beijing, China). Rabbit polyclonal antibody against GCN5, Bim, Egr1, active Caspase 3, mouse monoclonal antibody against *β*-galactosidase (*β*-Gal) (Cell Signaling Technology) and monoclonal antibody against NeurN (Millipore, Beijing, China) were used at a dilution 1:400, 1:100, 1:800, 1:100, 1:400 and 1:1000, respectively.

### RNA interference

Two GCN5 siRNAs, siGCN5a 5′-UGUUCGAGCUC UCAAAGAU-3′ and siGCN5b 5′-GCACCCACCUGAUGAAUCA-3′, and two Bim siRNAs, siBima 5′-CAACCAUUAUCUCAGUGCA-3′ and siBimb 5′-GACAGAGAAGGUGGACAAUUG-3′, non-sense control (NC) 5′-UUCUCCGAACGUGUCACGUTT-3′, the siEgr-1 5′-GGACUUAAAGGCUCUUAAU-3′ and shE2F1 targeting 5′-GCATTAGAGATCTCTTTGA-3′ were used. To detect the interference efficiency, these siRNAs and non-sense control siRNAs (NC) were transfected into rat C6 glioma cells by RNAiMax (Invitrogen) according to the manufacturer' s protocol. Forty-eight hours after transfection, cell lysates were harvested and processed for WB to detect the expression of these proteins.

In CGNs, DIV5 CGNs were transfected with NC and the siRNAs, together with pCMV-*β*-Gal by the calcium phosphate co-precipitation method.^28^ Forty-eight hours after transfection, cells were subjected to immunofluorescence and *β*-Gal was stained to mark the transfected cells. To determine the interference efficiency of the siRNAs, we referred to a method published by Cargnin *et al.*^63^ to quantify the rate of GCN5- or Bim-stained of all *β*-Gal-stained neurons. Briefly, images were acquired at × 20 magnification on confocal microscope using the same exposures. More than 100 cells transfected with siRNA or NC were taken. Each image was converted to 8 bit using the ImageJ software (NIH, Bethesda, MD, USA), and red channel (GCN5 or Bim staining) was adjusted to a 30–255 pixel threshold for counting GCN5- or Bim-stained of Gal-stained neurons.

For assessing the apoptosis rate affected by the siRNAs, EGFP was used to mark the transfected cells. After treatment, neurons were stained with Hoechst 33258 (5 *μ*g/ml), and apoptotic rate was determined by scoring the percentage of GFP-positive neuron population with pyknotic nuclei. Unbiased counting cells (~600 for each group) were scored blindly without knowledge of their previous treatment.

### Dual-luciferase reporter assays

The *bim*-luciferase (Luc) reporter, containing a 5.2 kb DNA fragment, was described previously.^29, 30^ For the dual-luciferase reporter assays, cells were transfected with 1 *μ*g of a luciferase reporter plasmid and 200 ng of the pRL–CMV *Renilla* luciferase reporter plasmid (Promega, Beijing, China). To test the effect of CPTH2 on the reporter, neurons were kept in conditioned media for 12 h after transfection and then transferred to serum-free media 25K containing CPTH2 (50 *μ*M) for 24 h. To observe the effect of siGCN5s on the reporter, neurons were kept in conditioned media for 48 h after transfection. *Firefly* luciferase activity was normalized to *Renilla* luciferase activity as reported previously.^37^

### IP and HAT activity assays

IP assays were performed as described previously.^64^ The lysed CGNs after 25K or 5K treatment or the homogenized sham or SAH brain samples were immunoprecipitated with 2 *μ*g GCN5 antibody or normal IgG and incubated with 30 *μ*l agarose hydrazide beads with protein A plus G (Merck Millipore, Beijing, China). A small part of immunocomplexes was subjected to WB analysis to test the efficiency of IP first, and the left was subjected to HAT activity assays by using the HAT Activity Assay Kit according to the manual (Sigma). Briefly, for each assay, water was added to the EP tube containing the left immunocomplexes to a final volume of 40 *μ*l and then 68 *μ*l assay mix was added containing: 2 × HAT Assay Buffer 50 *μ*l, HAT Substrate I 5 *μ*l, HAT Substrate II (mixing before use) and 5 *μ*l NADH Generating Enzyme 8 *μ*l. This was mixed by gently pipetting up and down and starting the reaction by incubating the tubes at 37 °C for 3 h. After centrifuging at 5000 r.p.m. for 2 min, the supernatant (100 *μ*l) was transferred to 96-well plate and sample was read in a spectrophotometer (Tecan Infinite, Männedorf, Switzerland) at 440 nm. HAT activity was expressed as the mean of O.D. values from at least three independent samples.

### SAH animal model

All experimental procedures were conducted under the guideline of Institutional Animal Care and Use Committee of Guangzhou Medical University. Rats had free access to food and water in a room with temperature maintained at 27 °C. Adult Sprague-Dawley rats (230–270 g) were purchased from the Animal Center of Sun Yat-sen University (Guangzhou, China). SAH model was induced by endovascular perforation method as previously described.^61^ Rats were anaesthetized with 10% chloral hydrate (0.35 ml/100 g, i.p.) and were positioned prone in a stereotactic frame (RWD Life Science Co., Ltd, Shenzhen, China). The left external and internal carotid arteries were exposed and a 3.0 monofilament nylon suture was inserted into the left internal carotid artery through the external carotid artery to perforate bifurcation of the anterior and middle cerebral arteries. Sham-operated rats underwent identical procedures except the perforation.

### Drug administration

Drug administration was performed as previously described.^61^ SAHA (Selleck Chemicals, Shanghai, China) was diluted in vehicle: sterile saline (11.25 *μ*l), PEG400 (11.25 *μ*l), and DMSO (2.5 *μ*l) at concentration of 5 mmol/l. The diluted SAHA (100 *μ*l/kg) or vehicle (100 *μ*l/kg) was administered into the right lateral ventricle 24 h before SAH was induced with the following coordinates relative to bregma: 1.5 mm posterior; 1.0 mm right lateral; 3.7 mm below the horizontal plane of bregma. The needle of a 50 *μ*l Hamilton syringe (RWD Life Science Co., Ltd) was inserted through a burr hole perforated on the skull. Automatically infused at a rate of 2 *μ*l/min and the needle was removed 5 min after completing an infusion.

### Experiment design and other methods

The detailed experiment designs and other methods used in this paper are listed in the Supplementary Data—Materials and Methods. The other methods, including measurement of brain water content, histone extraction from brain tissues, clinical evaluation, perfusion-fixation, TUNEL, slice preparation and cell counting, relative density analysis for the results of WB or RT-PCR, were performed as described previously.^41, 61, 65^

### Statistical analysis

The statistical software SPSS version 18.0 was used for statistical analysis (IBM Co., Armonk, NY, USA). Data were determined to be normally distributed before analysis. Student's two-tailed *t*-test was used for comparison between two different groups and ANOVA analysis was used with Fisher's LSD multiple-comparison test for multiple comparisons. All *P*-values <0.05 were considered statistically significant.
