# Supplementary material for: Loss of GCN5 leads to increased neuronal apoptosis by upregulating E2F1- and Egr-1-dependent BH3-only protein Bim
Source: Cell Death Dis. 2017 Jan 26;8(1):e2570–. doi: 10.1038/cddis.2016.465 (PMC5386373; doi:10.1038/cddis.2016.465)
Supplement: Supplementary Information [file cddis2016465x1.docx]

**Loss of GCN5 leads to increased neuronal apoptosis by upregulating E2F1- and Egr-1-dependent BH3-only protein Bim**

Yanna Wu^1, 2, #^, Shanshan Ma^3, 5, #^, Yong Xia^1, 2, #^, Yangpeng Lu^1, 2^, Shiyin Xiao^1, 2^, Yali Cao^1, 2^, Sidian Zhuang^1, 2^, Xiangpeng Tan^1, 2^, Qiang Fu^6^, Longchang Xie^1, 2^, Zhiming Li^4^, Zhongmin Yuan^1, 2, 3*^

**Supplementary data**

**
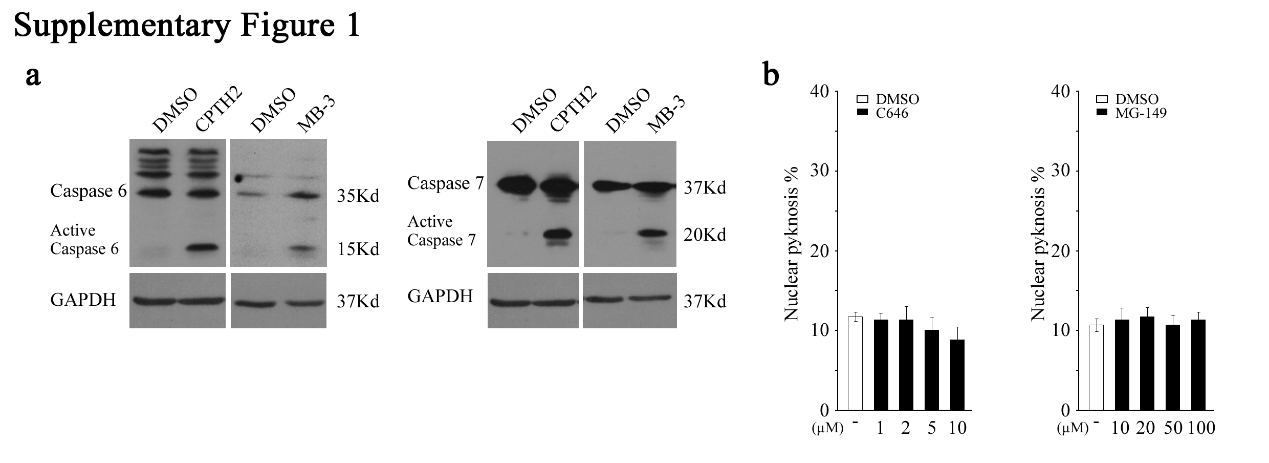
**

**Figure 1 Inhibition of GCN5 cause an activation of Caspase 6 and Caspase 7, but inhibition of p300 or Tip60 does not induce apoptosis** (**a**) CGNs treated with CPTH2 (50µM), MB-3 (150 µM) for 24 h were subjected to WB to determine the active Caspase 6, 7. (**b**) CGNs treated with C646 or MG-149 at the indicated doses for 24 h and apoptotic rate was determined by scoring the percentage of cells with nuclear pyknosis in total Hoechst-stained ones. Data with normal distribution were subjected to One-way ANOVA analysis and presented as means ± SEM (three experiments).


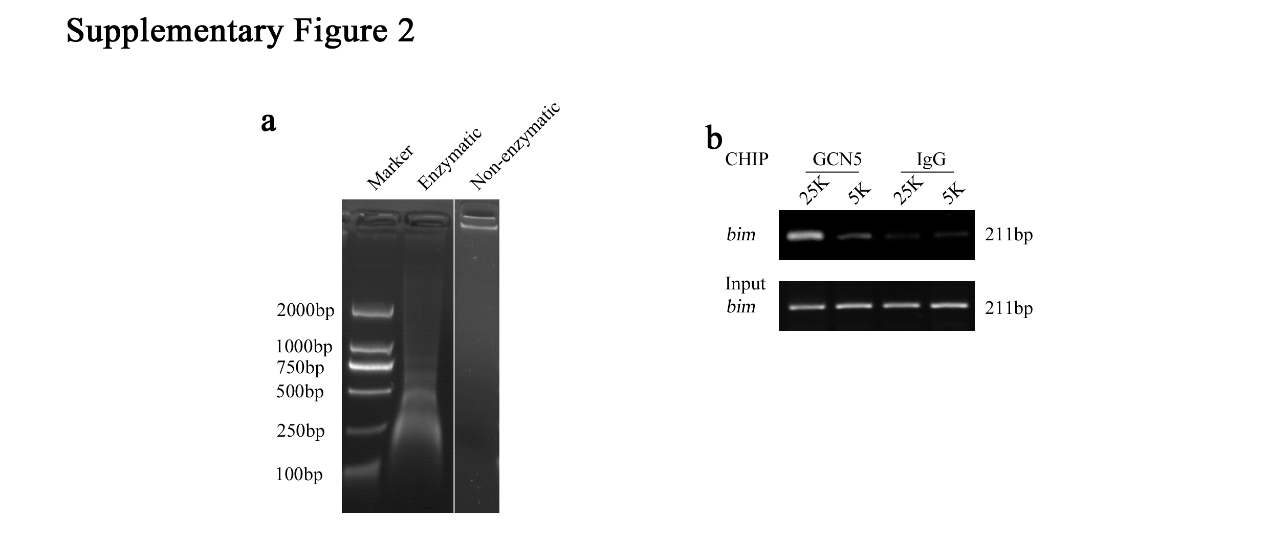


**Figure 2 potassium deprivation lower the binding activity of GCN5 to bim promoter** (**a**) CGNs treated with 25K or 5K for 4 h were subjected to CHIP assay and chromatin was cut by micrococcal nuclease into fragments of approximately 250-1000 bp. (**b**) The cleaved chromatin fragments were immunoprecipitated by anti-GCN5 antibody and then purified as templates to amplify the region of bim promoter by PCR. The part of chromatin fragments before immunoprecipitation was purified as templates to amplify bim promoter as a reference.


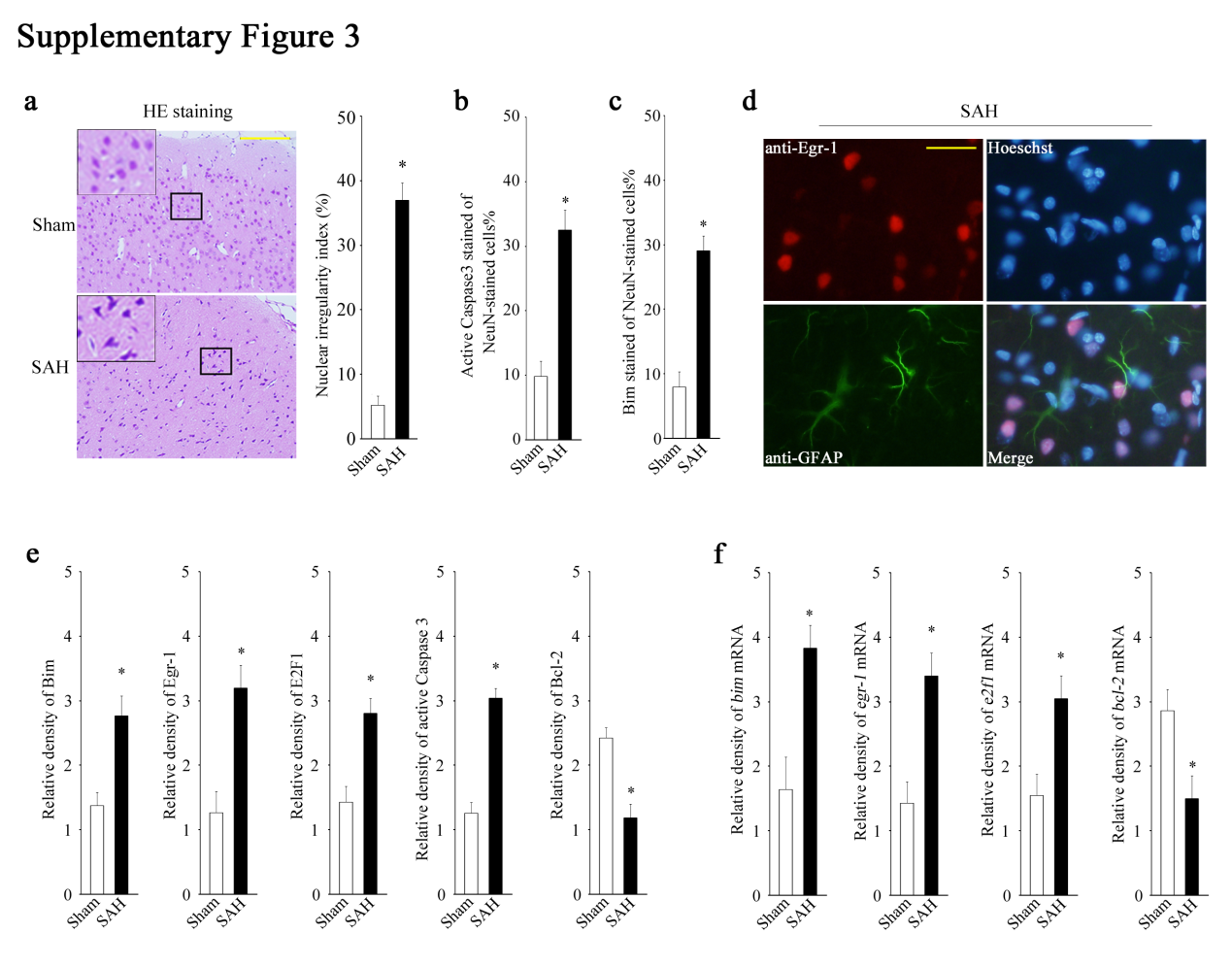


**Figure 3 SAH results in a typical neuronal apoptosis, concomitant with a transcriptional upregulation of Bim, E2F1 and Egr-1, but a downregulation of Bcl-2.** (**a**) At 24 h after SAH, H&E staining was performed to compare the difference of the nuclear irregularity rate between Sham and SAH groups. (**b, c**) The active caspase 3, Bim were detected by IF at 24h post SAH and their positive rates of all NeuN-stained cells were calculated. (**d**) No GCN5 was detected in GFAP-stained astrocyte cells in SAH rats by double staining at 24h post SAH. (**e**) Quantitative analysis of level of Bim, Egr-1, E2F1, the active Caspase 3 and Bcl-2 evaluated by Western blot. (**f**) Quantitative analysis of mRNA level of *bim, egr-1, e2f1,* and *bcl-2* evaluated by RT-PCR. Means ± SEM. *p*<0.05, n=6.


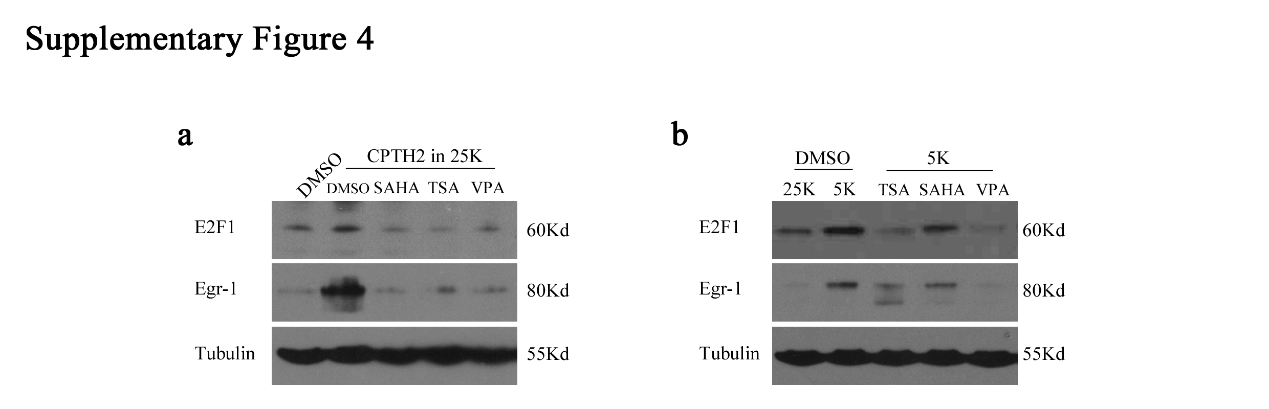


**Figure 4 HDACIs suppress E2F1 and Egr-1 expression induced by potassium deprivation or GCN5 inhibition.** (**a**) CGNs treated with DMSO, CPTH2 (50 µM) or CPTH2 together with SAHA (1 µM), TSA (0.5 µM), VPA (6 mM) for 24 h were subjected to WB to detect E2F1 and Egr-1. Tubulin was reprobed to verify equal loadings. (**b**) CGNs treated with 25K, 5K, or 5K together with SAHA (1 µM), TSA (0.5 µM), VPA (6 mM) for 12 h were subjected to WB to detect E2F1 and Egr-1. Tubulin was reprobed to verify equal loadings.


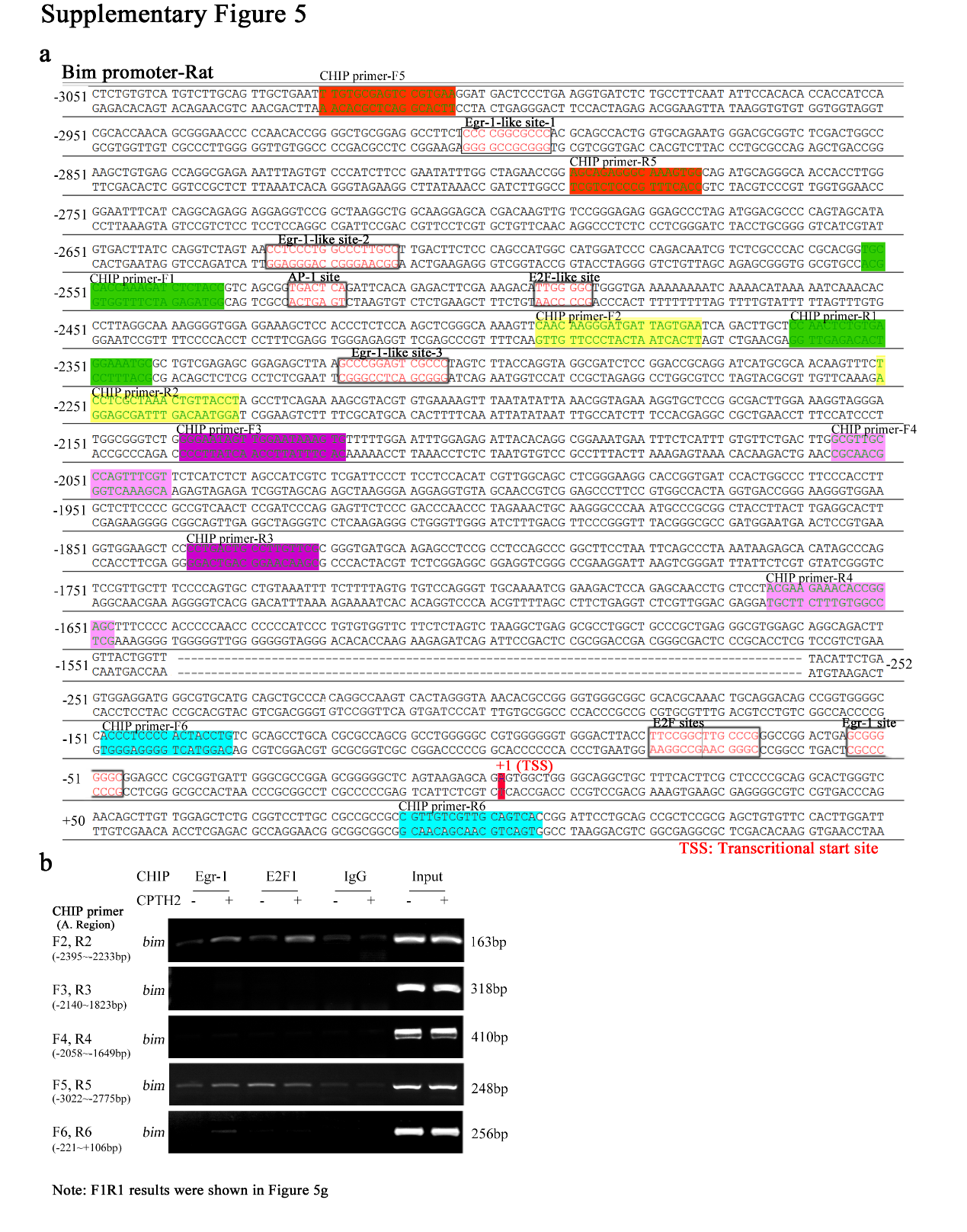


**Figure 5 The regions of bim promoter bound by Egr1 or E2F1 following GCN5 inhibition were determined by different primer pairs.** (**a**) The sequence of rat bim promoter was shown spanning -3051~+149bp. The position of each primer pair used in CHIP assay was indicated and the putative Egr-1 or E2F1 binding sites, the transcriptional start site (TSS) were marked. (**b**) The cleaved chromatin fragments were immunoprecipitated by anti-E2F1 or Egr-1 antibody and then purified as templates to amplify the region of bim promoter by using the different primer pairs indicated in (**a**).

Bim promoter-rat CHIP primer pairs

F1: 5' CAACAAGGGATGATTAGTGAA 3'

R1: 5' AGGTAACAGTTTAGCGAGGA 3'

F2: 5' GCAACAAGTTTCTCCTCGCTAA 3'

R2: 5' TGGGCAACGCCAAGTCA 3'

F3: 5' GGGAATAGTTGGAATAAAGTG 3'

R3: 5' CGAACAAGGCAGTCAGG 3'

F4: 5' GCGTTGCCCAGTTTCGT 3'

R4: 5' GCTCCGGTGTTTCTTCGT 3'

F5 5' TTGTGCGAGTCCGTGAA 3'

R5 5' CCACTTTGCCCTCTGCT 3'

F6 5′ ACCCTCCCCACTACCTG 3′

R6 5′ GTGACTGCAACGACAACG 3′

Note: F6R6 primer pair was used in the paper (Xie B, et al., J Neurosci, 2010).

**Supplementary-Materials and Methods**

**Experiment design**

***Experiment design 1*** – To perform neuronal apoptosis analysis and behavior and neurological score evaluation and the followed biochemistry analysis to test the expression of Bim, Egr-1, E2F1, Bcl-2 or Mcl-1 and the activity of GCN5, a total of 56 rats were used including 24 rats with sham and 33 rats with SAH (9 rats died). Died animals were excluded from further analysis. Both the sham rats and SAH rats were randomly divided into 4 subgroups according to the required assessments (1, brain water content; 2, IF, TUNEL and H&E staining; 3, WB and IP; 4, Extraction of histones and mRNA. n=6 for each subgroup). Clinical scores were recorded before Anesthesia and killed by ventricle perfusion at 24 h post SAH.

***Experiment design 2*** – To determine the role of HDAC inhibition by SAHA on neuronal apoptosis in rats with SAH and the possible mechanisms involved, a total 62 rats were used including two groups: SAH + vehicle group (32 rats, 8 rats died), SAH + SAHA group (31 rats, 7 rats died). The two groups were further randomly divided into 4 subgroups according to the required assessments (1, brain water content; 2, IF, TUNEL and H&E staining; 3, WB; 4, extraction of mRNA and histones. n = 6 for each subgroup).

**Measurement of Brain Water Content**

Brain edema was measured using the wet-weight/dry-weight method. Rats were decapitated at 24 h after SAH injury. The brains were rapidly removed and divided into the right and left hemispheres. Then, the brain samples were weighed before (wet weight) and after (dry weight) drying in an oven at 100 °C for 72 h. The brain water content was calculated as (wet weight − dry weight) /wet weight × 100 %.

**Histone extraction from brain tissues**

Briefly, 100mg brain tissues were to homogenized in 0.5 ml TEB buffer (PBS containing 0.5% Triton X 100, 2 mM PMSF and 0.02% NaN_3_) by 15-20 strokes. Then transfer mixture to a 2 ml vial and centrifuge at 10,000 rpm for 1 minute at 4°C. Remove supernatant and resuspend tissue pellet in 100µl extraction buffer (0.5N HCl + 10% glycerol) and incubate on ice for 30 minutes. Centrifuge at 12,000 rpm for 5 minutes at 4°C and remove the supernatant fraction to a new vial. Add 500 µl of acetone and leave at –20°C overnight. Centrifuge at 12,000 rpm for 5 minutes and air-dry the pellet. Dissolve the pellet in distilled 50µl water.

**Clinical evaluation**

Clinical scores were recorded before euthanasia based on the independent observations made by a veterinarian who was blind to the experimental groups. Three behavioral activity examinations (Table 1) including appetite, activity, and neurological deficits were used in the scoring methodology.

| Table 1 Behavior scores  Category Behavior Score  Appetite Finished meal 0  Left meal unfinished 1  Scarcely ate 2  Activity Walk and reach at least three corners of the cage 0  Walk with some stimulation 1  Almost always lying down 2  Deficits No deficits 0  Unstable walk 1  Impossible to walk 2 |
| --- |

**Perfusion-Fixation**

For histological or Immunofluorescence studies, animals were killed with the perfusion-fixation method. After the animals were deeply anesthetized with 10% chloral hydrate, perfusion-fixation was performed. The thorax was opened, a cannula was placed in the left ventricle, the descending thoracic aorta was clamped, and the right atrium was opened. Perfusion commenced with 500 ml of PBS at room temperature, followed by 500 ml of 4% buffered paraformaldehyde under a perfusion pressure of 120 cm H2O. After perfusion-fixation, the whole brain was removed and post-fixed in 4% paraformaldehyde followed by a subsequent immersion in 10%, 20%, 30% sucrose (weight/volume) for 24 hours each at 4 °C.

**TUNEL**

At 24 hours after sham or SAH-induction surgery, the rats were subjected to perfusion-fixation and the paraffin-embedded brain samples were cut into 4 µm sections. One slice from every 6 serial cuttings in each sample was selected, and altogether 4 slices were collected for TUNEL staining according to the manual of producer (Cat. No. : E607181, Sangon Biotech, Shanghai, China) after dewaxing and rehydration (procedure: Xylenes, 2 times, 2 min. each; 100% EtOH, 2 times, 2 min. each; 95% EtOH, 2 times, 2 min. each; 80% EtOH, 2 min; 75% EtOH, 2 min; 50% EtOH, 2 min. The nucleic was staining with Hoechst 33258 and photos were taken on microscope (IX-71, Olympus, Japan). The index of apoptosis was expressed as the ratio of TUNEL-positive cells to the total number of cells counted × 100% in each section counted in 6 cortical microscopic fields (at × 400 magnification). The final average percentage of TUNEL-positive cells of the 4 sections was regarded as the data for each sample.

**Slice preparation and cell counting**

One slice from every 6 serial cuttings in each segment was selected, and altogether 4-6 slices were collected for Immunofluorescence, TUNEL or H&E staining. The photos were taken on Confocal microscope (ZEISS, LSM 880) or fluorescence-inverted microscope (IX-71, Olympus) and the results were analyzed by 2 independent, experienced experimenters who were blinded to the grouping. All positive cells in each section were counted in 6 microscope fields (magnification × 400) throughout the identical regions of the studied brain.

**Relative density analysis for the results of WB or RT-PCR**

The expression changes of a gene in protein or mRNA levels between two experimental groups (animals) were determined by densitometry after normalization to tubulin, GAPDH or actin, accordingly. The quantification of band density was performed using ImageJ software (NIH, USA). All the results that need to be quantified were analyzed in the same PVDF member for Western blot or the same gel for RT-PCR. After normalization to the density of the corresponding band of a housekeeping gene product, each result was further normalized to the minimum one of them to get a normalized ratio. The differences between means of the normalized ratios from two experimental groups were determined.
